# Genome reconstruction of white spot syndrome virus (WSSV) from archival Davidson’s-fixed paraffin embedded shrimp (*Penaeus vannamei*) tissue

**DOI:** 10.1038/s41598-020-70435-x

**Published:** 2020-08-10

**Authors:** Roberto Cruz-Flores, Hung N. Mai, Siddhartha Kanrar, Luis Fernando Aranguren Caro, Arun K. Dhar

**Affiliations:** Aquaculture Pathology Laboratory, School of Animal and Comparative Biomedical Sciences, The University of Arizona, Building 90, Tucson, AZ USA

**Keywords:** Biological techniques, Microbiology techniques, Diseases, Infectious diseases, Viral infection

## Abstract

Formalin-fixed paraffin-embedded (FFPE) tissues are a priceless resource for diagnostic laboratories worldwide. However, DNA extracted from these tissues is often not optimal for most downstream molecular analysis due to fragmentation and chemical modification. In this study, the complete genome of white spot syndrome virus (WSSV) was reconstructed from ~ 2-year-old archived Davidson’s-fixed paraffin-embedded (DFPE) shrimp tissue using Next Generation Sequencing (NGS). A histological analysis was performed on archived DFPE shrimp tissue and a sample showing a high level of WSSV infection was selected for molecular analysis. The viral infection was further confirmed by molecular methods. DNA isolated from DFPE and fresh frozen (FF) tissues were sequenced by NGS. The complete genome reconstruction of WSSV (~ 305 kbp) was achieved from both DFPE and FF tissue. Single nucleotide polymorphisms, insertion and deletions were compared between the genomes. Thirty-eight mutations were identified in the WSSV genomes from the DFPE and FF that differed from the reference genome. This is the first study that has successfully sequenced the complete genome of a virus of over 300 kbp from archival DFPE tissue. These findings demonstrate that DFPE shrimp tissue represents an invaluable resource for prospective and retrospective studies, evolutionary studies and opens avenues for pathogen discovery.

## Introduction

Formalin-fixed paraffin-embedded (FFPE) archived tissues in human and animal pathology laboratories are an invaluable resource for clinical research, genetic studies and pathogen discovery^1–3^. In human cancer research, the utilization of preserved material from tissue banks has helped circumvent the need to rely upon frozen tissue and has enabled the reanalysis of samples from diverse clinical trials^1,2,4^. The most common and suitable way to store tissues over prolonged periods of time is to fix them in formaldehyde and embed them in paraffin. However, the extraction of high quality nucleic acids from fixed tissues that is required for Next Generation Sequencing (NGS) has proven challenging.

Formalin is the mostly widely used fixative that allows the preservation of tissues at room temperature and maintains the structure of cells and other components^5,6^. However, formalin fixation presents one major drawback as it cross-links macromolecules, including DNA and RNA, which greatly complicates the extraction of intact DNA and RNA from FFPE tissues^5,6^. Formalin fixation also causes hydrolysis of phosphodiester bonds, leading to varying degrees of DNA fragmentation^6,7^. In addition, the degradation of nucleic acids increases during storage depending on the pH value of the fixative^8^. Histopathological evaluation of shrimp tissues requires the fixation of tissue in Davidson´s fixative (alcohol, formalin and glacial acetic acid)^9^. This type of fixation is fundamental in histopathology studies involving shrimp tissue since acetic acid enables the softening of the chitinous exoskeleton of shrimp. The major drawback of this fixative is its acidic pH (~ 3.5–4) which causes further degradation of nucleic acids due to induced acid hydrolysis and acidophilic endogenous ribonuclease activity^10,11^.

In human clinical research, many researchers have successfully extracted nucleic acids from FFPE tissue for PCR-based amplification work despite the degradation of nucleic acids and thereby giving them access to a previously untapped resource. However, it must be clarified that in most cases the successful use of FFPE tissue for molecular analysis depends largely on how the sample was fixed (temperature, time, pH). In cancer research FFPE tissues have been extensively used for genome sequencing of tumor tissues for copy-number and mutation-analysis, expression profiles, screening for mutational hotspots, single-cell sequencing and genome sequencing from Laser Capture Microdissected cells^1–3,12–14^. FFPE tissues have been used in pathogen discovery and uncovering novel genetic features in pathogen genomes. For example, the Spanish Influenza Pandemic Virus was reconstructed from FFPE tissue from 1918^15^. The virus species involved in 1918 pandemics showed large differences to the contemporary human influenza H1N1 strain^15^. Recently, FFPE tissue has been used for the detection and discovery of a novel rotavirus^5^. In another recently published retrospective study, FFPE tissue was used to sequence the RNA genome of ~ 15 kb length of the Newcastle disease virus (NDV) that naturally infects many avian species^16^. The study revealed the continuous evolution and previously unrecognized genetic diversity in NDV^16^.

The first study of the use of FFPE material in aquatic organisms dates back to 1995 when Krafft et al.^17^ used fixed tissue to detect morbillivirus in lung tissue of bottlenose dolphins. In finfish, mollusk and crustacean pathology, so far no attempt has been made to explore the feasibility of using FFPE/DFPE tissues for any retrospective genetic studies or pathogen discovery. In shrimp aquaculture, existing as well as emerging diseases are a threat to a sustainable growth of the industry worldwide. Outbreaks of diseases in shrimp aquaculture cause major economic losses to shrimp farmers directly, and indirectly impact the lives and livelihood of those who depend of shrimp farming especially in developing nations with large coastal boundaries. There is an urgent need to understand the origin and evolution of pathogens in shrimp aquaculture to prevent epizootics that are becoming more common than ever. Archived Davidson's-fixed paraffin-embedded (DFPE) tissues in the Aquaculture Pathology Laboratory of The University of Arizona are an untapped invaluable resource for pathogen discovery, metagenomic and evolutionary studies to understand the origin, evolution and spread of shrimp pathogens worldwide. In this study, we demonstrated the feasibility of using DFPE tissue in pathogen discovery by reconstructing the complete genome of a large dsDNA-containing virus, white spot syndrome virus (WSSV), with a genome size of  ~ 305 kbp from DFPE tissues. To our knowledge, this is the first report of genome reconstruction of such a large genome from archived tissue for any virus known to infect humans, animals or plants. This study shows the utility of DFPE tissues in shrimp pathology research, opens avenues for novel pathogen discovery and enables us to address questions related to the origin and evolution of shrimp viral pathogens that continue to cause catastrophic losses to farmers globally.

## Results

### Selection of Davidson's-fixed paraffin embedded blocks

**Figure 1:**
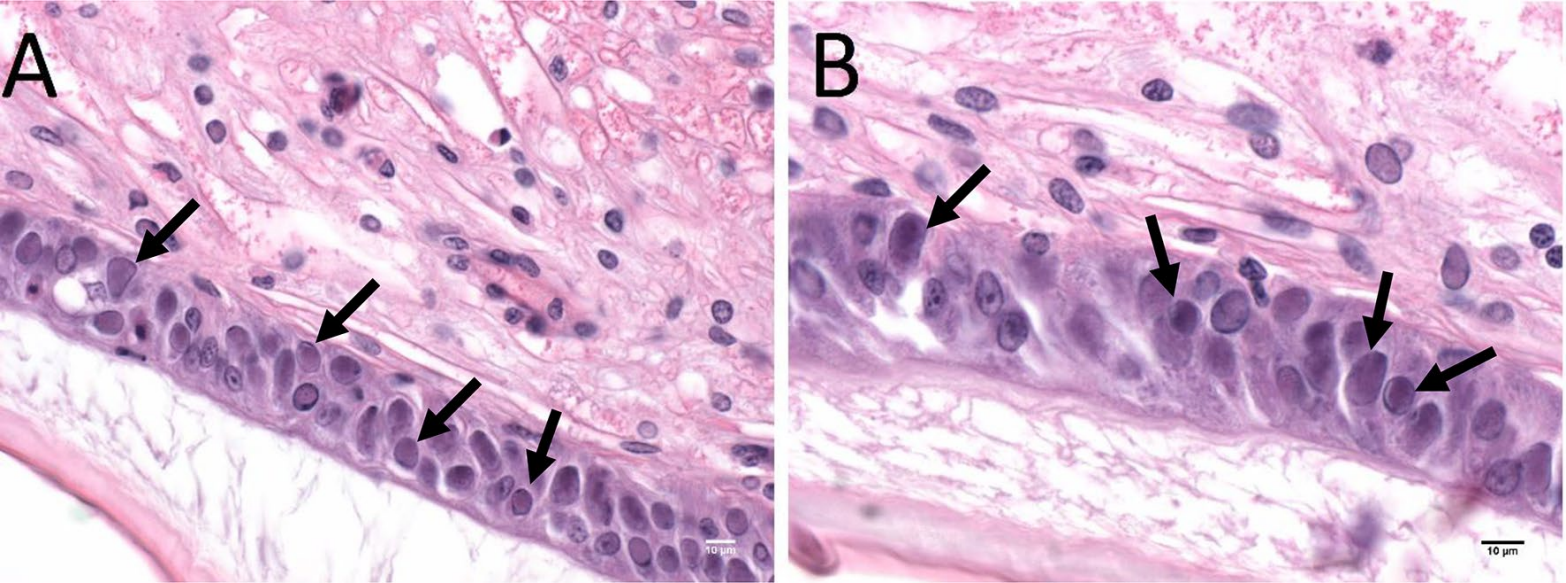
Intranuclear basophilic inclusion bodies of white spot syndrome virus in the cuticular epithelia of *P. vannamei* (sample no. 17-702 A11). (**A**) Cross sections of the cuticular epithelia displaying numerous WSSV inclusion bodies indicated by the black arrows (×40 magnification). In (**B**) the WSSV inclusion bodies are shown at a higher magnification (×60 magnification). Scale bars = 10 µm.

Histopathological evaluation of the experimentally infected *Penaeus vannamei* shrimp revealed a severe infection graded as G3–G4 in all tissues examined. A representative histology section showing the intranuclear inclusions in the gut epithelial cells of *P. vannamei* that are pathognomonic of WSSV infection is shown in Fig. 1. Based on the histopathology evaluation, a sample, No. 17-702 A11 was selected for NGS.

### DNA quality, quantity and PCR

Using the commercial kit FFPE DNA Purification Kit (NORGEN BIOTEK CORP), DNA was eluted twice from each sample. The first DNA elution obtained from the DFPE shrimp tissue yielded a higher concentration that ranged from 139.7–76.5 ng/µl. The second elution yielded a lower concentration that ranged from 23.6–11.9 ng/µl. A summary of the DNA concentrations is provided in Supplementary Table 1. All the samples tested positive by qPCR (Cycle threshold value shown in Supplementary Table 1) and nested PCR (Supplementary Fig. 1).

### First sequencing of WSSV from DFPE shrimp tissue

The initial NGS attempt to determine the feasibility to generate sequence reads from DFPE shrimp (Aquaculture Pathology Laboratory Research from 2017 Case no. 17-702) tissue provided 2,247,970 reads in a PE 2 × 300 bp format. A total 29,107 unique reads, approximately 1.3% reads, were mapped to the WSSV reference genome (GenBank accession number: AF332093) generating a consensus sequence of 305,111 bp covering almost the complete genome of WSSV. Additionally, a histogram of read size distribution is presented in Supplementary Fig. 2.

### Sequence analysis of the WSSV genome from DFPE shrimp tissue and annotation

**Figure 2:**
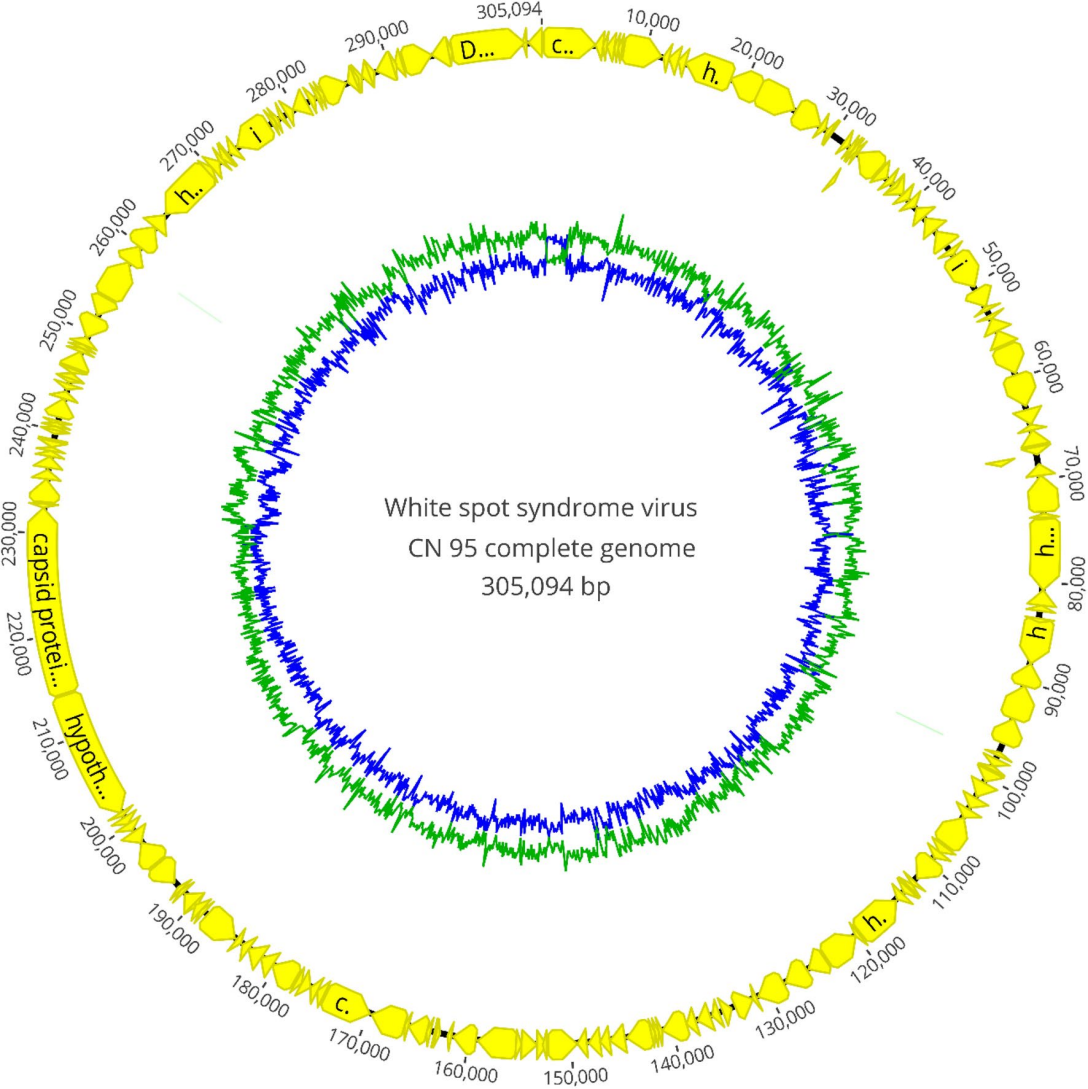
Complete genome sequence of white spot syndrome virus reconstructed from Davidson’s-fixed paraffin-embedded shrimp tissue. A total of 193 DNA coding sequences were annotated using RAST and Geneious Prime. The blue internal line represents the GC content and the green internal line represents the AT content. The open reading frames indicated by yellow shaded boxes on both orientations are shown in the genome.

A total of 308,724,322 million reads were generated from the second round of sequencing in a PE 2 × 150 bp format. From the total of reads, 3,056,486 unique reads (approximately 1.0% reads) were mapped to the WSSV China reference genome and generated complete coverage of the entire genome with a mean coverage of 1,550.6. The maximum and minimum coverage obtained were 5,219 and 317, respectively. The reconstructed WSSV genome was 305,094 bp and presented a pairwise identity of 99.90% with the genome of the China reference strain. A total of 193 DNA coding sequences were annotated using RAST and Geneious Prime (Fig. 2). Additionally, a histogram of read size distribution is presented in Supplementary Fig. 2.

### Sequence analysis of the WSSV genome from FF shrimp tissue and annotation

A total of 128,299,466 million reads were generated in a PE 2 × 150 bp format. From the total of reads, 5,346,756 unique reads (approximately 4.1% reads) were mapped to the WSSV China reference genome and generated complete coverage of the entire genome with a mean coverage of 2,637.9. The maximum and minimum coverage obtained were 11,614 and 558, respectively. The generated genome sequence was 305 kbp and presented a pairwise identity of 99.90% with the genome sequence of the China reference strain deposited in the NCBI database (GenBank accession number AF332093). The WSSV genome from FF shrimp tissue presented a pairwise identity of 99.99% with the WSSV genome reconstructed from the DFPE tissue. A total of 193 DNA coding sequences were annotated using RAST and Geneious Prime. Additionally, a histogram of read size distribution is presented in Supplementary Fig. 2.

### Confirmation of SNPs and sequence variations

**Figure 3:**
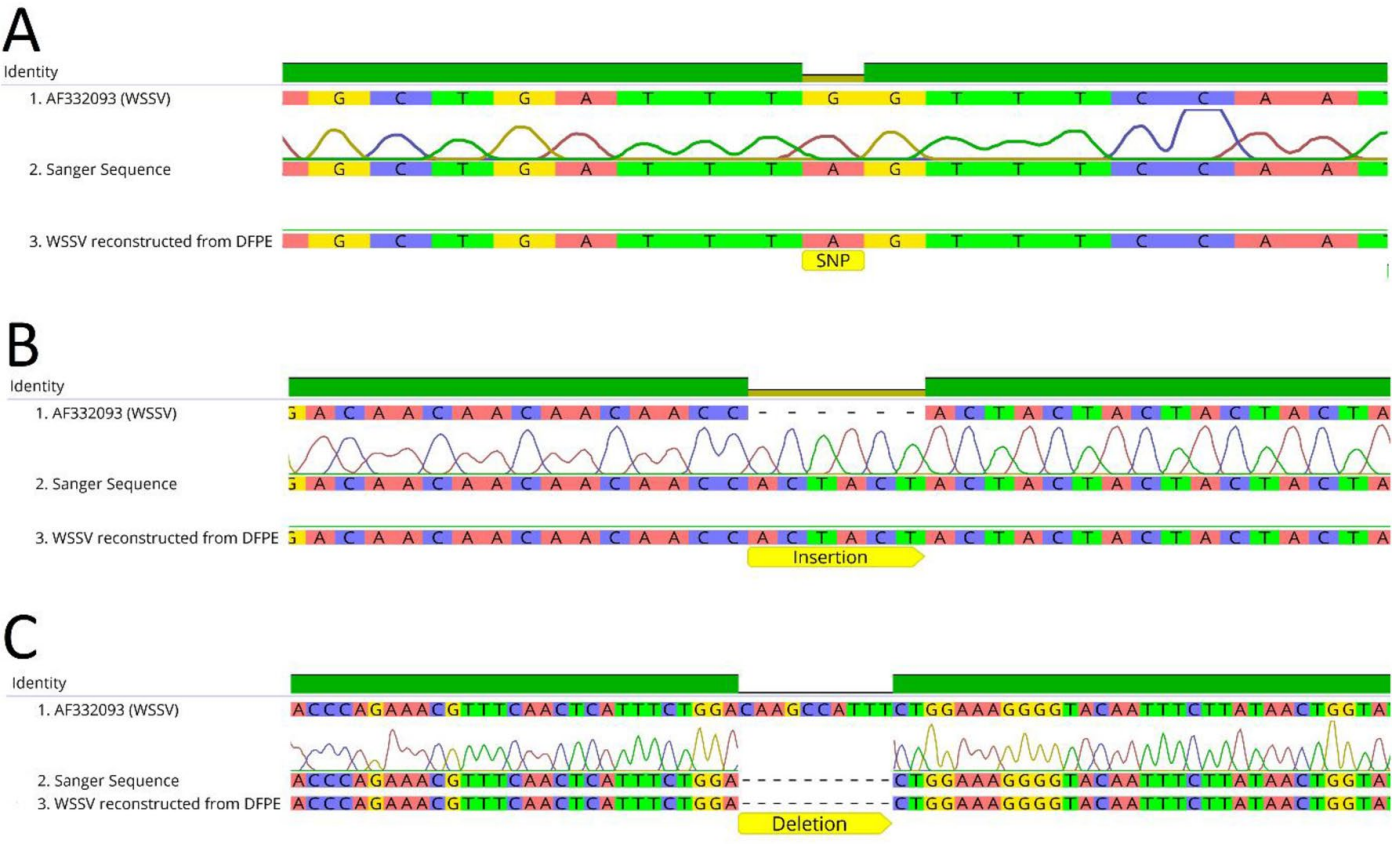
Confirmation of the sequence variations observed in the white spot syndrome virus (WSSV) genome reconstructed from DFPE tissue. The figure shows single nucleotide polymorphism (SNP) (**A**), insertion (**B**) and deletion (**C**). The WSSV reference sequence (1) is shown on top, the Sanger sequence is shown in the middle (2) and the WSSV sequence reconstructed from DFPE (3) tissue is shown in the bottom. (**A**) Represents the SNP located in position 25, 783. (**B**) Represents the insertion located in positions 263,177–263,183. (**C**) Represents the deletion located at 240,504.

The WSSV genome sequence obtained from DFPE tissue sample were aligned with GenBank sequence of the WSSV China reference strain and the SNPs were identified. A total of 38 sequences variations that include SNPs, deletions or insertions were detected. The genomic regions that contained the SNPs and sequence variations were amplified by PCR, sequenced and aligned with the reference WSSV genome and the WSSV genome reconstructed from DFPE tissue (Table 1, Fig. 3). The SNPs and sequence variations were confirmed when the Sanger sequence and the DFPE derived sequence matched. All 38 of the mutations found in the WSSV genome reconstructed from DPFE were confirmed by Sanger sequencing.

**Table 1:** Confirmation of sequence variations, single nucleotide polymorphisms (SNPs), insertions and deletions between the genome sequence of white spot syndrome virus reference strain (AF332093), the WSSV genome reconstructed from Davidson’s-fixed paraffin-embedded tissue and fresh frozen tissue.

Location	DFPE NGS	FF NGS	Sanger sequence	Result	Type of nucleotide variation
10,721	SNP	SNP	SNP	Confirmed	10,721A > G
17,698	SNP	SNP	SNP	Confirmed	17,698T > C
25,783	SNP	SNP	SNP	Confirmed	25,783G > A
29,539	SNP	SNP	SNP	Confirmed	29,539A > T
30,570	Deletion	Deletion	Deletion	Confirmed	30,569_30,72delCC
32,725	SNP	SNP	SNP	Confirmed	32,727G > A
37,497	SNP	SNP	SNP	Confirmed	37,499G > A
54,067	SNP	SNP	SNP	Confirmed	54,067G > A
55,001	SNP	SNP	SNP	Confirmed	55,003C > T
59,536	Deletion	Deletion	Deletion	Confirmed	59,537_59,540delCC
63,406	SNP	Deletion	SNP	Confirmed	63,410A > T
100,420	SNP	SNP	SNP	Confirmed	100,427T > A
102,021	Deletion	SNP	Deletion	Confirmed	102,027_102,030delCC
113,604	SNP	SNP	SNP	Confirmed	113,614C > A
124,885	SNP	SNP	SNP	Confirmed	124,895C > T
141,081	Deletion	Deletion	Deletion	Confirmed	141,090_141,094delCTT
186,344	Deletion	Deletion	Deletion	Confirmed	186,360_186,356delCCT
190,834	SNP	SNP	SNP	Confirmed	190,850A > G
197,909	Insertion	Insertion	Insertion	Confirmed	197,924_197,925insT
226,074	SNP	SNP	SNP	Confirmed	226,089C > T
236,920	SNP	SNP	SNP	Confirmed	236,935T > C
239,361	SNP	SNP	SNP	Confirmed	239,376A > C
239,385	SNP	SNP	SNP	Confirmed	239,400T > C
239,391	SNP	SNP	SNP	Confirmed	239,406G > T
239,394	SNP	SNP	SNP	Confirmed	239,408C > G
239,650	SNP	SNP	SNP	Confirmed	239,667T > G
240,478	Deletion	Deletion	Deletion	Confirmed	240,503_240,514delCAAGCCATTT
240,003	Deletion	Deletion	Deletion	Confirmed	240,017-24019delC
240,164	Deletion	Deletion	Deletion	Confirmed	240,179-240,190delACAAGCCATTT
240,256	SNP	SNP	SNP	Confirmed	240,282A > G
240,266	SNP	SNP	SNP	Confirmed	240,292C > G
240, 288	SNP	SNP	SNP	Confirmed	240,314T > C
241,149	Insertion	Insertion	Insertion	Confirmed	241,184_241,185insA
261,974	SNP	SNP	SNP	Confirmed	262,009G > T
263,193	Insertion	Insertion	Insertion	Confirmed	263,227_263,228insCTACTA
276,936	SNP	SNP	SNP	Confirmed	276,965C > A
296,010	Insertion	Insertion	Insertion	Confirmed	296,038_296,039insC
303,439	Insertion	Insertion	Insertion	Confirmed	303,466_303,467insAGC

The location of the nucleotide variations, the type of modifications detected by next generation sequencing (NGS) and Sanger sequencing are presented in the table. The location of the SNPs, deletions and insertions are shown in respect to the reference WSSV genome (AF332093).

## Discussion

Detection and characterization of novel viruses is frequently hampered by the lack of properly stored materials^5^. For the retrospective identification of viruses associated with disease outbreaks, often only formalin-fixed paraffin-embedded (FFPE) tissue samples are available^5^. Retrieving genomic information from FFPE has always been a challenge but the availability of genome sequencing technologies such as NGS has now made it possible to reconstruct genetic information from archived samples. In the case of viral diseases of shrimp, often the etiologic agent was identified long after the disease was initially reported and spread within and across countries worldwide. While samples originally collected during disease outbreaks were used for histopathological and ultrastructural studies to elucidate possible etiologic agent, those samples were almost never used for genetic characterization of the pathogen associated with the disease. As a result, it remains unknown how the pathogen evolved during the time frame from when the disease outbreaks were initially reported vs. when the genomic characterization of the pathogen was accomplished. For example, the Infectious Hypodermal and Hematopoietic Necrosis Virus (IHHNV) was initially detected in 1981 by Lightner et al.^18^ however, the genetic characterization of the virus was carried out 19 years later by Shike et al.^19^. IHHNV was the first shrimp virus for which the genome sequence was determined^19^. Considering a high rate of nucleotide substitution (1.39 × 10^–4^ substitutions/site/year)^20^ of IHNNV, it is possible that the strain that caused massive mortalities of blue shrimp (*P. stylirostris*) in the early 80’s in Mexico and later in the rest of the Americas is different from the strains that have been characterized later. Another interesting fact that supports this hypothesis is that the current strains of IHNNV do not cause mortalities or major histological alterations in *P. vannamei* and *P. stylirostris* shrimp and no major epizootics have been attributed to IHHNV in recent years^20,21^. This could be due to accumulation of mutations in the IHHNV genome and/or development of host resistant/tolerance over time. Upon reconstructing the WSSV genome from DFPE tissues, we have shown that the genetic characterization of pathogens containing large genomes is possible from archived fixed tissue (DFPE) and this opens the door for future retrospective studies to better understand the genomic properties of pathogens from the past that once caused mass mortalities but causes little to no mass mortality anymore. These studies would enable to better understand the evolution of host–pathogen interactions not only in shrimp but also in viruses infecting other animals and humans.

In this study, the DNA extracted from the DFPE tissue were used for pathogen detection via PCR and qPCR. WSSV was successfully detected by qPCR and nested PCR following OIE-recommended (Paris, France) protocols and WSSV genomic fragments ranging 69 bp (for real-time PCR) to 1,477 bp (for 1st step of the nested PCR) were amplified. However, it is important to mention that for the nested PCR protocol the increase in the amount of template DNA was key to obtaining amplification in all samples (N = 7). In preliminary assays, amplification was only obtained in two samples (17-702 A6 and 17-702 A7) when using > 130 ng of DNA per reaction. All the fragments designed to amplify the areas where the SNP where located ranged between 100–250 bp and thus fell within the size range of qPCR and nested PCR diagnostics. A previous published study involving human housekeeping genes by Ludyga et al.^8^ have shown that products between 100–300 bp can be reliably amplified from FFPE. The study by these authors also showed that the amplifiable fragment size decreases with storage time with the maximum amplifiable fragment decreasing from 687 bp from samples from the year 2000 to 129 bp from samples from 1971^8^. Our results show that a relative short storage time (~ 2 years) of DFPE shrimp tissue, as used in this study, can provide DNA of sufficient quality that can be used to amplify DNA fragments of almost 1,500 bp. In addition, these results suggest that Davidson’s fixative is not as damaging to nucleic acids as previously postulated by Hasson et al.^11^ and that DNA from DFPE shrimp tissue does not undergo sever degradation in short storage times (~ 2 years). However, we should underscore that some degradation does occur, as observed from the percentage of virus mapping reads where the FF sample had a high percentage (~ 4%) in comparison to the DFPE samples (~ 1–1.3%) and our inability to amplify large PCR products at lower DNA concentrations.

Single-nucleotide polymorphism changes in bacterial genomes can cause significant changes in phenotype, including antibiotic resistance and virulence, therefore detecting them within metagenomes is vital^22^. The same can be said about SNPs in viral genomes where single nucleotide changes can have a profound effect on replication and pathogenicity. In Taura syndrome virus (TSV) of shrimp, it was shown that a single nucleotide mutation changed the predicted tertiary structure of the RNA-dependent-RNA polymerase in a highly virulent strain compared to less virulent strain^23^. The error rates for some NGS data sets generated by Illumina technologies are very low: a rate 0.0021 (errors per base)^22^. Despite the low error rate, it was critical to confirm that the detected SNPs, insertions and deletions were present in the WSSV genome reconstructed from DFPE and were not sequencing errors. By amplifying, and sequencing the regions where the variations were located, we were able to confirm all the SNPs, insertions and deletions. This results further highlight the robustness of this methodology since it proves that small sequence variations can be efficiently detected from DFPE tissue and it underscores its value for retrospective phylogenetic analysis.

In recent years, the availability of novel genome sequencing technologies have increased the chance and speed of detection of unknown viruses in samples collected from humans and animals. In particular NGS played an important role in the discovery and characterization of many novel viruses^5,24–26^. Next generation sequencing using DNA isolated from FFPE tissue enabled pathogen detection, identification of endogenous viral elements, genome sequencing, exome and transcriptome sequencing in animals and humans^1–5,7,12,14,16,27,28^. Although FFPE tissues have been used to detect known viral sequences, the application of FFPE tissues for detection of novel viruses is very limited. Recently, Bodewes et al.^5^ showed that sequence-independent amplification in combination with NGS can be used to detect sequences of known and unknown viruses in herring gull and ferrets, although with relatively low sensitivity. The findings of Bodewes et al.^5^ indicate that NGS from FFPE is a viable approach to detect known DNA (Adenovirus) and RNA (influenza A/H1N1) viruses, and unknown RNA viruses (novel herring gull rotavirus). Our results confirm that NGS from DNA extracted from DFPE tissue is also a viable approach to detect know viral sequences. However, our results show this approach is robust and can generate enough data to sequence very large viral genomes with a very high coverage. Furthermore, our results suggest that with sufficient data even the sequencing of complete bacterial genomes from this type of samples might be possible.

Unlike plant and human virology, shrimp virology is a relatively newly emerged field of virology. The first shrimp viral disease was reported only about 50 years ago (*Baculovirus penaei*)^29^ and the first shrimp virus was sequenced about 20 years ago (IHHNV)^19^. However, as shrimp aquaculture has evolved from a subsistence level of farming to a major industry providing jobs to millions of people around the world directly and indirectly especially in countries with large coastal boundaries, viral diseases poses a serious threat to the sustainable growth of this nascent industry. As of now, viral disease prevention through biosecurity and early disease diagnosis remain as corner stones to mitigate losses in shrimp aquaculture. Since these diseases primarily spread through the movement of infected broodstock and post-larvae across countries and continents, it is critical to understand how these pathogens evolve in new environment as virus-infected animals are moved across continents and how naïve host adapt to new pathogens. The ability to reconstruct DNA viral genomes as large as 300 kbp size from DFPE tissues shows the feasibility to generate baseline genetic data from archived tissue and determine how pathogens have evolved over time. To our knowledge, this is the first study that shows the feasibility of using NGS as a viable option for genetic characterization of shrimp pathogens and potentially discovering novel pathogens from samples stored in pathology laboratories worldwide.

## Materials and methods

### Generation of the WSSV infected shrimp samples

Specific Pathogen Free (SPF) Pacific white shrimp (*P. vannamei*) were experimentally challenged via oral route with WSSV (China isolate CN95 strain). Briefly, previously minced and frozen China isolate WSSV (CN 95) positive shrimp was fed to the tank at a rate of 5% bodyweight for a single feeding. Beginning the following day, the animals were maintained on a commercially pelleted shrimp diet. Moribund shrimp were collected at 3 days post infection and parallel tissues were fixed in Davidson´s fixative and liquid nitrogen for histopathological examination and molecular biology work, respectively (University of Arizona-Aquaculture Pathology Laboratory Research from 2017 Case no. 17-702). The samples were ~ 2 years old at the time of the of the analysis.

### Histopathology and selection of Davidson’s-fixed paraffin-embedded blocks

Histopathological evaluations were performed on slides from the experimentally infected *P. vannamei* from Case 17-702 (N = 7). The severity of the WSSV infection was graded based on a semi-quantitative scale that ranges from Grade 0 to Grade 4 following a previous publication^9^. While Grade 0 shows no signs of infection, Grade 1 show signs of infection by the pathogen but at levels that may be below those needed for clinical disease, Grade 2 moderate signs of infection shown by number and severity of pathogen caused lesions, Grade 3 moderate to high signs of disease shown by number and severity of pathogen caused lesions and Grade 4 high numbers of pathogen caused lesions and tissue destruction. Paraffin blocks that derived from histological sections that presented a Grade 4 infection level were selected for DNA extraction (Fig. 1).

### DNA extraction, quantification and PCR

DNA was extracted using the commercial kit FFPE DNA Purification Kit (NORGEN BIOTEK CORP) in accordance with the manufacturer’s recommendations with some modifications. During the deparaffinization step, the xylene washes were doubled, and the pellet was air dried for 20 min. Finally, during the lysate preparation step, the incubation at 90 °C was increased from 1 to 1 h 15 min. Two elution’s were obtained from each sample. Additionally, from the samples fixed in liquid nitrogen DNA was extracted using the Genomic DNA isolation kit (NORGEN BIOTEK CORP) following the manufacturer’s instructions. The quantity and quality of the DNA was determined using a NanoDrop 2000. The presence of WSSV was further confirmed by qPCR and nested-PCR following published protocols^30,31^. For the nested-PCR protocol published by Lo et al.^30^ one modification was made, the input volume of DNA was increased to 2.5 µl (191.25–349.25 ng/per reaction).

### Next generation sequencing (NGS)

To test the feasibility of performing NGS using DNA isolated from DFPE shrimp tissue, we conducted two rounds of NGS. The first sample was sequenced using an Illumina MiSeq System (PE 2 × 300 bp) (Illumina). Once we determined it was possible to efficiently sequence DNA from archived DFPE tissues using an Illumina MiSeq System, we sequenced two additional samples. The second round of sequencing was done using an Illumina HiSeq 2500 System (PE 2X150 bp) (Illumina) to generate a more robust data set. DNA extracted from both DFPE tissue and FF tissue were sent for NGS at OmegaBioservices, Norcross, GA. Library for the DNA samples were generated at OmegaBioservices using the Library Kit, KAPA Hyper prep for WGS (Roche). For the DNA extracted from the DFPE tissue the fragmentation step prior to library generation was avoided since the isolated DNA was already fragmented.

### Mapping and annotation

The DNA reads were paired and duplicate reads were removed using the Dedupe plugin in Geneious Prime^32^. DNA reads from the WSSV shrimp tissue (DFPE and FF) were checked for quality and trimmed before being mapped to the China WSSV reference genome (GenBank accession number: AF332093) using Geneious Prime (Biomatters)^32^. The Geneious mapper was used for the mapping analysis and the setting were set to detect structural varients^32^. The WSSV isolate of the APL originated from China and hence the China WSSV reference genome was utilized, however, it is unknown if they represent the same strain. The mean coverage of each based was calculated. The contigs generated from the mapping were annotated using the RAST Server and Geneious Prime^32,33^. The WSSV complete genome reconstructed from DFPE (GenBank accession: MN840357) was submitted to GenBank. The MAUVE software was used to perform whole genome alignments and comparisons^34^. The genomes of WSSV obtained from the DFPE tissue, FF tissue and the WSSV reference genome were compared to identify differences among the sequences.

### Confirmation of single nucleotide polymorphisms (SNPs) and sequence variations

To confirm the sequence variations observed between the WSSV China reference genome and the WSSV genome reconstructed from the DFPE tissues, primers were designed to amplify the regions flanking the SNPs, deletions and insertions. SNPs located in the repeat regions were not analyzed. Primers were designed using Geneious Prime. The nucleotide sequence of primers designed to amplify the regions showing variations are presented in Table 2. Each PCR amplification was carried out in a total volume of 25 µl containing 1 µl of template DNA (50–100 ng/µl), 12.5 µl of DreamTaq Hot Start Green PCR Master Mix (ThermoFisher) and 350 nM of each primer pair (1-F/R-WSSV to 31-F/R-WSSV), as shown in Table 2. The PCR conditions consisted of an initial denaturation at 95 °C for 2 min, followed by 45 cycles at 95 °C for 10 s, 60 °C for 10 s and 72 °C for 10 s with a final elongation step at 72 °C for 2 min. The PCR products were run on a 2% agarose gel and were visualized on a GelDoc XR + (Bio-Rad). The amplicons were sequenced at the University of Arizona Genetics Core. The sequences were analyzed using Geneious Prime. To confirm that the mutations (single nucleotide polymorphisms, insertions and deletions) were present, the WSSV reference genome sequence (AF332093), the DFPE sequence and the sanger sequence were aligned using the Geneious aligner.

**Table 2:** The nucleotide sequence and location of the primers flanking the single nucleotide polymorphism (SNPs), deletions and insertion regions in WSSV genome constructed from the Davidson's-fixed paraffin-embedded shrimp tissue.

Location	Primer name	Sequence	Type of mutation
10,622–10,641	1-F-WSSV	GACCACACCAGCCCTAAAGG	SNP
10,796–10,815	1-R-WSSV	TTCGATTTGGGTCCTCCGTC	
17,628–17,649	2-F-WSSV	CAATGGGCATAACCTTGTTGGA	SNP
17,753–17,777	2-R-WSSV	AGCGTTCTTCAAGATCAATAGGAGA	
25,727–25,746	3-F-WSSV	ATGCTGGCTCTCGATTCGTT	SNP
25,870–25,889	3-R-WSSV	AAGGCCCACTTAATCCAGCG	
29,447–29,466	4-F-WSSV	GGTCAGCCGTGTTCCAGAAA	SNP
29,584–29,603	4-R-WSSV	GGTCGACCCAACGTCAGATT	
30,491–30,510	5-F-WSSV	TGGTTGTTGCTGCTGAGAGA	Deletion
30,626–30,646	5-R-WSSV	ACCAGATGTGAGTCAAACCGT	
32,621–32,641	6-F-WSSV	TCACCCTTCATCTCCATCTCA	SNP
32,753–32,778	6-R-WSSV	TGGTATACATTTCTAGACCCTCTCTG	
37,439–37,460	7-F-WSSV	TCAACACCCATGATTTTAGTTT	SNP
37,573–37,592	7-R-WSSV	CCGTTTGCTTGGCGGTAAAA	
53,956–53,975	8-F-WSSV	TTCTCCACAACGTTGACGGG	SNP
54,090–54,111	8-R-WSSV	ACCGTTAAACCAAGAAACAGCA	
54,919–54,939	9-F-WSSV	GTTGGTTGGTTGGTTGTGGAC	SNP
55,048–55,068	9-R-WSSV	AGATATGGCGCAAGAAGAGGG	
59,444–59,463	10-F-WSSV	CGTAGGTGTCGGGGCTAAAT	Deletion
59,586–59,605	10-R-WSSV	CGACCGTCGATGTCTTCACA	
63,301–63,320	11-F-WSSV	GTTTGCTGTGGTGGTTACGG	SNP
63,466–63,485	11-R-WSSV	AGACTTTGGCTCCATCACGG	
100,328–100,352	12-F-WSSV	TGAGTGGGTTTCTTTAGTATGTGGA	SNP
100,468–100,487	12-R-WSSV	TCCAGGTTAACTTGCCAGCC	
101,924–101,944	13-F-WSSV	GGTGTATTTGAGACCGTCTGC	Deletion
102,071–102,090	13-R-WSSV	CGAGGGAATGATGCTGTGGT	
113,525–113,544	14-F-WSSV	AGCACGAAAGGGTCCACAAA	SNP
113,670–113,689	14-R-WSSV	TCTCCCAATCTCCTCCAGCT	
124,782–124,806	15-F-WSSV	AGACTAATATAACGTCATGGCCTGT	SNP
124,927–124,946	15-R-WSSV	TACGGTTGGTCACTGCTGTT	
140,983–141,009	16-F-WSSV	TTTTCATTTCCTTCCTTTTTAAAGTGT	Deletion
141,120–141,140	16-R-WSSV	CCTTAGCAGGGACCTAACCAG	
186,246–186,267	17-F-WSSV	TGGTTGATTATCGTCGTCTTCT	Deletion
186,395–186,414	17-R-WSSV	TGCTGGTGGAGTATGTGCTG	
190,745–190,764	18-F-WSSV	TACACACTTGGAACCCACCC	SNP
190,879–190,899	18-R-WSSV	ACCTTCTTCTTTTGCACGTCT	
197,808–197,827	19-F-WSSV	ACGCCATGGATGAACTTCTT	SNP
197,937–197,957	19-R-WSSV	TGGTTGCACTGTCATAACACT	
226,006–226,032	20-F-WSSV	AGAAGAAACTGTTAATAGTGGTATGGT	SNP
226,155–226,175	20-R-WSSV	TGATCAAGAGCTGGTCGACTC	
236,835–236,854	21-F-WSSV	TTGAAGGAGGTGACAGGTGC	SNP
236,995–237,014	21-R-WSSV	ACAAGTGAGCTGCATGATCA	
239,296–239,319	22-F-WSSV	TCTGGTGCATTATTTCTGGTACCA	SNPs
239,452–239,473	22-R-WSSV	AGGAAGTTTCACTCCATCTCCA	
239,574–239,593	23-F-WSSV	TGGACCACTCCCATTTCTGG	SNPs
239,718–239,737	23-R-WSSV	GTCGCTCCACTGGTAGTGTT	
240,064–240,083	24-F-WSSV	ACATGAACACATGAGGCGGT	Deletion
240,228–240,248	24-R-WSSV	TCGACCCAATGTCAGATTGCA	
240,352–240,379	25-F-WSSV	TATAGTACTCCGTAGCCAACATATACAC	Deletion
240,933–240,960	25-R-WSSV	AAAAATTTTTCTGCGTCACTCGAGTTTA	
241,045–241,072	26-F-WSSV	CCACATCTGCGTCATACATTATATTTCC	Insertion
241,217–241,244	26-R-WSSV	ACAGATTTTGTCCATATGATGATTCTCT	
261,880–261,899	27-F-WSSV	GGGACAATAAGCGCAACACA	SNP
262,013–262,032	27-R-WSSV	GGGCAATTTCTTCCAGTGCG	
263,092–263,113	28-F-WSSV	AGAAGTAGAAGTTGCGCTACCT	Insertion
263,254–263-275	28-R-WSSV	ACTGCCAAAGATTTCTGGTTCA	
276,850–276,869	29-F-WSSV	TCTGTATCAGCAGCAGCAGC	SNP
276,998–277,017	29-R-WSSV	AATGTTGGGCCGTATCCGTT	
295,904–295,923	30-F-WSSV	AACCCTAACAATGGTGTGCC	Insertion
296,038–296,059	30-R-WSSV	ACACATATCTCATCGCACGTCT	
303,342–303,363	31-F-WSSV	GCTGCATGTCTATCTTGTGTTT	Insertion
303,519–303,538	31-R-WSSV	ACGACCATGGGCTGTAGAAA	

## Supplementary information

Supplementary Information.

## Data Availability

All the data generated during this study are available from the corresponding author upon request.
