# Supplementary material for: Genome reconstruction of white spot syndrome virus (WSSV) from archival Davidson’s-fixed paraffin embedded shrimp (Penaeus vannamei) tissue
Source: Sci Rep. 2020 Aug 10;10:13425. doi: 10.1038/s41598-020-70435-x (PMC7417530; doi:10.1038/s41598-020-70435-x)
Supplement: Supplementary file 1 — Supplementary Information. [file 41598_2020_70435_MOESM1_ESM.docx]

Genome reconstruction of white spot syndrome virus (WSSV) from archival Davidson’s-fixed paraffin embedded shrimp (*Penaeus vannamei*) tissue

Roberto Cruz-Flores^a^, Hung N. Mai^a^, Siddhartha Kanrar^a^, Luis Fernando Aranguren Caro^a^, Arun K. Dhar^a^

^a^ Aquaculture Pathology Laboratory, School of Animal and Comparative Biomedical Sciences, Building 90, The University of Arizona, Tucson, Arizona, USA

*Corresponding author: Arun K. Dhar, email: [adhar@email.arizona.edu](mailto:adhar@email.arizona.edu)

**Supplementary files**

Table 1. DNA concentration, 260/280 ratio and 260/230 ratio for the DNA extracted from Davidson-fixed paraffin-embedded shrimp tissue infected with WSSV.

| **Sample** | **Elution** | **Concentration (ng/µl)** | **260/280** | **260/230** | **Cycle threshold** |
| --- | --- | --- | --- | --- | --- |
| 17-702 A4 | 1 | 97.0 | 1.79 | 2.09 | 21.14 |
| 17-702 A4 | 2 | 12.9 | 1.67 | 1.25 | 23.65 |
| 17-702 A5 | 1 | 76.5 | 1.80 | 1.78 | 23.73 |
| 17-702 A5 | 2 | 18.8 | 1.76 | 1.32 | 23.98 |
| 17-702 A6 | 1 | 130.3 | 1.77 | 2.02 | 20.41 |
| 17-702 A6 | 2 | 19.9 | 1.74 | 1.75 | 21.55 |
| 17-702 A7 | 1 | 139.7 | 1.80 | 1.77 | 25.00 |
| 17-702 A7 | 2 | 21.6 | 1.76 | 1.18 | 25.79 |
| 17-702 A8 | 1 | 83.0 | 1.80 | 1.63 | 19.31 |
| 17-702 A8 | 2 | 11.9 | 1.88 | 1.33 | 20.03 |
| 17-702 A9 | 1 | 115.2 | 1.81 | 1.05 | 22.19 |
| 17-702 A9 | 2 | 16.1 | 1.67 | 0.85 | 24.11 |
| 17-702 A11 | 1 | 120.6 | 1.91 | 2.03 | 17.98 |
| 17-702 A11 | 2 | 23.6 | 1.86 | 1.67 | 19.21 |


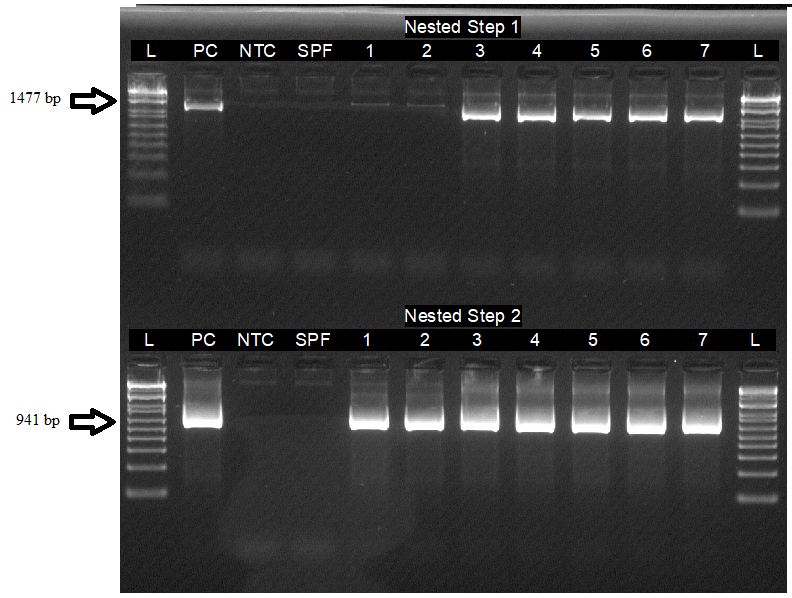


Figure 1. Gel electrophoresis of nested PCR amplicons of White spot syndrome virus amplified using the DNA samples extracted from DFPE shrimp tissue (case 17-702). All samples 17-702 A4, 17-702 A5, 17-702 A6, 17-702 A7, 17-702 A8, 17-702 A9 and 17-702 were positive for WSSV for the second step and show a clear 941 bp amplicon. L (Ladder, Invitrogen 1Kb Plus), PC (Positive control), NTC (No template control), SPF (Specific pathogen free), 1 (17-702 A4), 2 (17-702 A5), 3 (17-702 A6), 4 (17-702 A7), 5 (17-702 A8), 6 (17-702 A9) and 7 (17-702 A11).


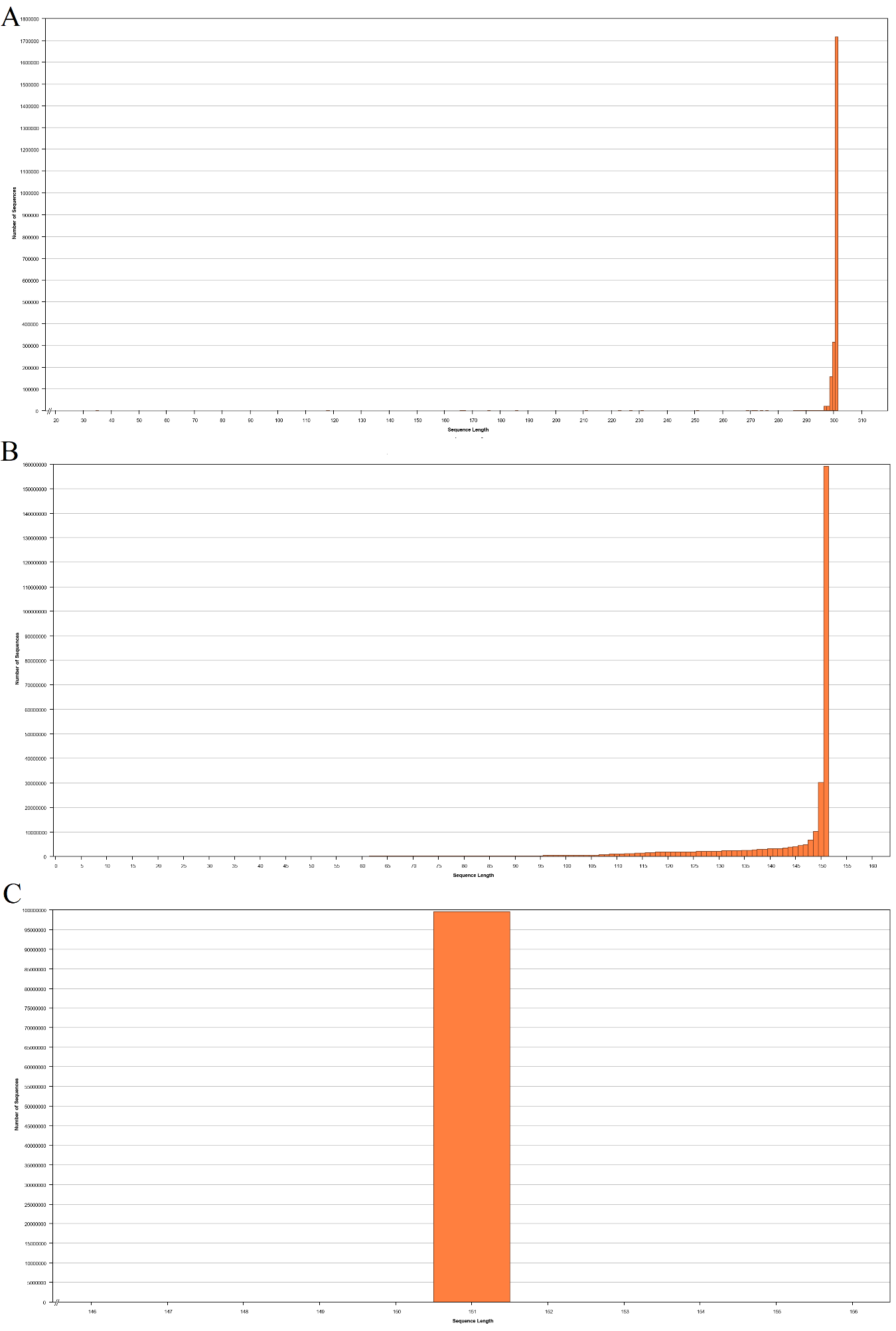


Figure 2. Read size distribution after duplicate read removal for first WSSV DFPE sequencing, the second WSSV DFPE sequencing and the WSSV FF sequencing. (A) First WSSV DFPE sequencing yielded 2,247,970, the mean sequence length of 300.8, Std Dev 0.7, a maximum sequence length of 301 and a minimum sequence length of 35. (B) The second DFPE WSSV sequencing yielded 308,724,322, the mean sequence length was 142.6, Std Dev 15.9, a maximum sequence length of 151 and a minimum sequence length of 0. The WSSV FF sequencing yielded 128,299,466, the mean sequence length was 151, Std Dev 0.
